# Psychosis Polyrisk Score (PPS) for the Detection of Individuals At-Risk and the Prediction of Their Outcomes

**DOI:** 10.3389/fpsyt.2019.00174

**Published:** 2019-04-17

**Authors:** Dominic Oliver, Joaquim Radua, Abraham Reichenberg, Rudolf Uher, Paolo Fusar-Poli

**Affiliations:** ^1^Early Psychosis: Interventions and Clinical Detection (EPIC) Lab, Department of Psychosis Studies, Institute of Psychiatry, Psychology and Neuroscience, King's College London, London, United Kingdom; ^2^OASIS Service, South London and the Maudsley NHS Foundation Trust, London, United Kingdom; ^3^Institut d'Investigacions Biomèdiques August Pi i Sunyer (IDIBAPS), CIBERSAM, Barcelona, Spain; ^4^Department of Clinical Neuroscience, Centre for Psychiatry Research, Karolinska Institutet, Stockholm, Sweden; ^5^Institute of Psychiatry, Psychology and Neuroscience, King's College London, London, United Kingdom; ^6^Department of Psychiatry, Icahn School of Medicine at Mount Sinai, New York, NY, United States; ^7^Department of Preventive Medicine, Icahn School of Medicine at Mount Sinai, New York, NY, United States; ^8^Frieman Brain Institute, Icahn School of Medicine at Mount Sinai, New York, NY, United States; ^9^Department of Psychiatry, Dalhousie University, Halifax, NS, Canada; ^10^Maudsley Biomedical Research Centre, South London and Maudsley NHS Foundation Trust, National Institute for Health Research, London, United Kingdom; ^11^Department of Brain and Behavioral Sciences, University of Pavia, Pavia, Italy

**Keywords:** schizophrenia, clinical high risk, risk, psychosis, prediction, environment, polygenic risk, genetics

## Abstract

Primary prevention in individuals at Clinical High Risk for psychosis (CHR-P) can ameliorate the course of psychotic disorders. Further advancements of knowledge have been slowed by the standstill of the field, which is mostly attributed to its epidemiological weakness. The latter, in turn, underlies the limited identification power of at-risk individuals and the relatively modest ability of CHR-P interviews to rule-in a state of risk for psychosis. In the first part, this perspective review discusses these limitations and traces a new approach to overcome them. Theoretical concepts to support a Psychosis Polyrisk Score (PPS) integrating genetic and non-genetic risk and protective factors for psychosis are presented. The PPS hinges on recent findings indicating that risk enrichment in CHR-P samples is accounted for by the accumulation of non-genetic factors such as: parental and sociodemographic risk factors, perinatal risk factors, later risk factors, and antecedents. In the second part of this perspective review we present a prototype of a PPS encompassing core predictors beyond genetics. The PPS prototype may be piloted in the next generation of CHR-P research and combined with genetic information to refine the detection of individuals at-risk of psychosis and the prediction of their outcomes, and ultimately advance clinical research in this field.

## Highlights

– Research in individuals at Clinical High Risk for Psychosis is at a standstill.
– Limitations include low detection power and suboptimal prognostic accuracy.
– Psychosis Polyrisk Scores (PPS) have the potential to improve the detection of at-risk individuals.
– Psychosis Polyrisk Scores (PPS) have the potential to optimize the prediction of psychosis.

## Introduction

Psychotic disorders such as schizophrenia are among the world's leading causes of disability from psychiatric disorders (1). Under standard care, outcomes of psychosis are relatively poor (2). The implementation of early intervention services for patients experiencing their first episode of illness may improve the course of the disorder (3). However, recent meta-analytical evidence indicates there is no robust evidence that these services can effectively prevent psychotic relapse (3) or reduce the duration of untreated psychosis (4). Thus, there are high expectations that primary prevention in individuals who have not yet experienced the disorder can ameliorate its course (5). In clinical practice, such a strategy has been limited to indicated prevention that is offered to individuals at Clinical High Risk for Psychosis [CHR-P (6)]. The definitions and description of specific CHR-P instruments have been fully presented in previous publications (7). In brief, the CHR-P state defines a condition of liability toward the development of incident psychotic disorders, but not of any other incident non-psychotic mental disorder (8, 9). CHR-P research has allowed the study of the factors that predate the onset of psychosis and experimental therapeutics to be trialed for the prevention of psychosis (e.g., omega-3 fatty acids (10, 11). However, its impact on improving the outcomes of psychotic disorders has been constrained by significant limitations. The present perspective review originates from a critical analysis of these limitations and confronts this in two sections. In the first part, it traces a new conceptual avenue for future research—tackling the above constraints by formulating the theoretical groundwork. In the second part, a practical prototype of a new prognostic tool is introduced to inform the future development of more efficient strategies to detect individuals at-risk for psychosis and the prediction of their outcomes.

## Methods

For the first part, a critical review of the past literature was conducted. Relevant articles were retrieved through international databases (PubMed, books, meetings, abstracts, electronic guidelines, and international conferences) and critically reviewed by the authors of the paper. Subsequently, results were presented after reaching a consensus and were summarized through illustrative tables and figures. This review is not following a systematic literature search, data extraction, or reporting approach, since its ultimate aim is to provide a conceptual perspective of the field. In the second part, we applied the concepts refined through the critical literature search to the field of psychosis prediction. We thus operationalize a Psychosis Polyrisk Score (PPS) and present it. Simulation analyses complemented our approach to provide some initial feasibility and prognostic values associated with the use of the PPS. Further details of the operationalization of the PPS and how simulation analyses were conducted can be seen in section “Psychosis Polyrisk Score (PPS) Prototype”.

## Conceptual Review of the Limitations of the Clinical High Risk State for Psychosis

### The Epidemiological Weakness of the Clinical High Risk State for Psychosis

**Figure 1:**
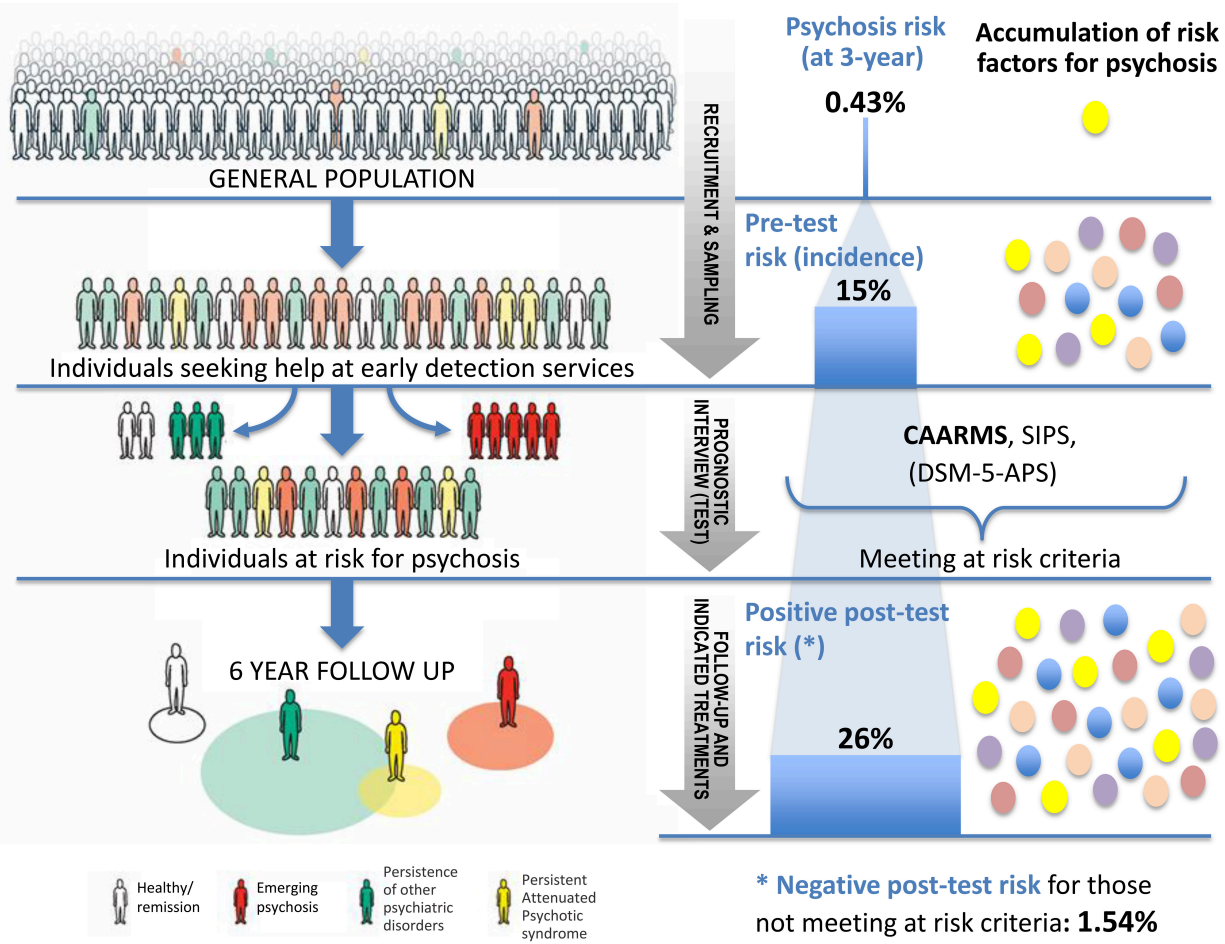
Individuals seeking help at specialized psychosis early-detection clinics have a higher (pre-test) risk of developing psychosis [15% at 3 years (14)] than the general population (0.43% at 3 years) (17). Those who will meet the clinical high risk for psychosis (CHR-P) criteria at the prognostic interview (Comprehensive Assessment of At-risk Mental States [CAARMS]) will have only a modest increase in their (post-test) level of risk for psychosis (1.7-fold, from 15 to 26%). Those not meeting the CHR-P criteria (18) will have a substantial decrease in their (post-test) risk (10-fold, from 15 to 1.56%).

**Figure 2:**
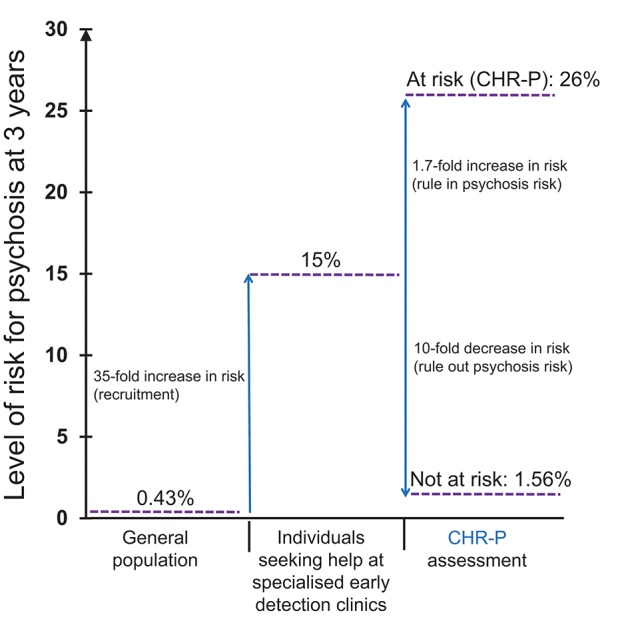
Sampling procedure for individuals at clinical high risk for psychosis (CHR-P) (14). Idiosyncratic recruitment strategies that are characterized by heterogeneous sampling biases (convenience and judgmental sampling) result in the accumulation of various risk factors for psychosis and differential level of enrichment of psychosis risk. The figure is based on the data reported in Fusar-Poli et al. (17), Fusar-Poli (21), and Rutigliano et al. (22). CAARMS, Comprehensive Assessment of At Risk Mental States; SIPS, Structured Interviews for Psychosis-Risk Syndromes; DSM-5-APS, Diagnostic and Statistical Manual, 5th Edition, Attenuated Psychosis Syndrome. Adapted from: Fusar-Poli et al. (12).

**Figure 3:**
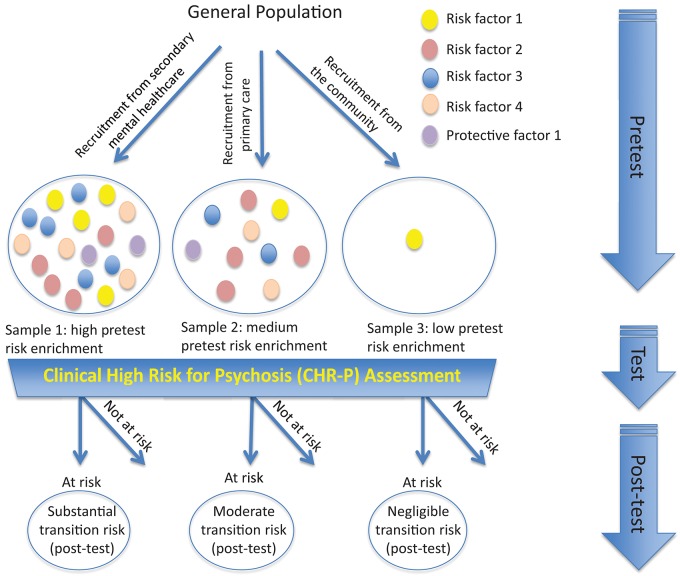
Effect of sampling biases in the CHR-P paradigm. Idiosyncratic recruitment strategies lead to differential accumulation of risk factors for psychosis across samples undergoing CHR-P assessment. For example, recruiting from secondary mental health care (sample 1) is associated with high risk enrichment for psychosis (also termed “pretest” risk) compared to recruiting from the general practitioners (sample 2), while no or little psychosis-risk enrichment is observed if the sample is randomly selected from the general population (sample 3). Applying the CHR-P interviews to these samples discriminates between those at-risk for psychosis and those not at-risk (post-test risk). However, the actual (post-test) transition to psychosis that is observed at follow-up largely depends on the overall level of accumulation of risk factors for psychosis during the risk enrichment phase and only in minor part on the results (i.e., testing at-risk or not at-risk) of the CHR-P assessment itself.

To illustrate the epidemiological weakness associated with the CHR-P paradigm we present data from our experience of detecting and providing clinical care to these individuals in South London (12). First, by using validated population-level prediction tools (e.g., www.psymaptic.org), we estimated the annualized incidence of psychotic disorders in the local general population (13). The recruitment of individuals who may be at CHR-P for psychosis is primarily based on unstructured selection and sampling strategies that are based on clinician's suspicion of psychosis risk (14) and on help-seeking behaviors (15). Therefore, the way these individuals are sampled will determine their level of accumulation of risk factors for psychosis. For example, when individuals undergoing a CHR-P assessment are recruited from mental health services, they accumulate several risk factors for the disorder (16) which increase their level of risk to 15% at 3-years, compared to the 0.43% 3-year risk in the local age-matched general population (12, 17) (Figure 1). This level of risk is also termed as “pre-test risk,” because it is ascertained in the whole group of people undergoing a CHR-P assessment before the results of the assessment itself are known (19). Therefore, the level of risk of samples undergoing a CHR-P assessment does not reflect the level of risk of the general population, but it is substantially higher: from 0.43% at 3-year to 15% at 3-year (about 35-fold-higher). Once these individuals complete a CHR-P assessment, they will be predicted to have a certain post-test risk of developing psychosis or not. Thus, pre-test and post-test risks of psychosis index an individual's likelihood of developing psychosis before and after the results of the CHR-P assessment are known, respectively (19). It follows that the value of a test will depend on its ability to alter (increase or decrease) a pre-test probability of a target condition into a post-test probability that will influence a clinical management decision (20). When these individuals with a 15% pretest risk at 3-year are assessed (tested), those who will meet CHR-P criteria will have a 26% risk of developing psychosis at 3-year (1.7-fold increase) and those who will not meet the CHR-P criteria will have a 1.56% risk of developing psychosis at 3-year (10-fold decrease) (Figure 2). The relationship between the risk enrichment accounted by the recruitment step (pre-test) and diagnostic assessment step (post-test) (19) is illustrated in specific charts (Nomograms) that have been externally validated (23). It confirms that once individuals are recruited for undergoing a CHR-P assessment, there is only limited prognostic gain in meeting the CHR-P criteria (i.e., testing positive to the interview), while there is some prognostic gain in not meeting the CHR-P criteria (i.e., testing negative to the interview). In other words, the CHR-P tools are quite good at ruling out a state of psychosis risk but not very good at ruling it in; they can only be clinically meaningful when applied to samples that have been risk-enriched. When different CHR-P instruments (7) or even the DSM-5 category of Attenuated Psychosis Syndrome, -which is not psychometric-based and therefore not strictly speaking a CHR-P instrument- are applied to these samples, they produce comparable prognostic performance (24, 25). As shown in Figure 3, the actual risk of developing psychosis in CHR-P samples is thus largely dependent on the way individuals are recruited for the assessment and on their pre-test risk enrichment (14, 17). The additional challenge is that recruitment strategies for individuals undergoing CHR-P assessment and therefore pre-test risk enrichment are highly heterogeneous, idiosyncratic and poorly standardizable (14). This results in a high variance of risk enrichment across samples undergoing CHR-P assessment [meta-analytical 48-months risk of psychosis 95%CIs 0.09–0.24 (14), Figure 3]. Therefore, CHR-P samples that undergo distinct psychosis risk enrichment pathways are hardly comparable as they are likely to have different profiles of risk factors (26, 27). These notions have both clinical and research implications. On a clinical level, the variable risk enrichment of CHR-P samples may amplify variations in patients' clinical needs and limit the provision of standard clinical care. On a research level, CHR-P samples with little risk enrichment or heterogeneous risk profiles may lead to negative findings in neurobiological studies (28) or even in preventative trials (29–31). Overall, because of these points, the key limitation of the CHR-P paradigm is currently that of substantial epidemiological weakness (27, 32).

### Idiosyncratic Accumulation of Risk Factors in Individuals With a Clinical High Risk State for Psychosis

Risk factors contributing to the psychosis risk enrichment observed in CHR-P samples are not entirely known. A recent meta-analysis has summarized the available evidence across 54 putative risk factors investigated in CHR-P samples, in comparison to controls (16). Astoundingly, there are no existing studies on the association between genetic or epigenetic risk factors and the CHR-P state. Although family history for psychosis is partially embedded in CHR-P criteria, its predictive significance within the CHR population is questionable. A recent collaborative meta-analysis has found that CHR-P individuals with a familial history of psychosis do not have an enhanced risk of developing psychosis within 4 years follow-up, compared to controls (33). Essentially, the above meta-analysis showed that CHR-P subjects are more likely to show obstetric complications, tobacco use, physical inactivity, childhood trauma, high perceived stress, childhood and adolescent low functioning, affective comorbidities, male gender, single status, unemployment, and low educational level as compared to controls (16). Overall, this study suggests that risk enrichment of CHR-P samples can be attributed to demographic and environmental risk factors like childhood trauma, adverse life events and affective dysfunction. The differential combination of risk/protective factors in each CHR-P individual is likely to account for the distinct clinical outcomes observed in these samples: psychosis onset, recovery, or disability (6).

### Limited Detection Power

An additional problem is that the risk profiles observed in CHR-P individuals who will develop psychosis may not be representative of a prototypical first episode of psychosis. CHR-P individuals who later transition to psychosis represent only about 5% of first episode patients within secondary mental health care (34). This suggests there is limited detection power for at-risk cases and inefficient recruitment strategies (5). Such a limitation is substantial, undermining the significance of the entire paradigm. Although CHR-P interviews are particularly good at ruling out psychosis, only a minority of individuals are referred for a full CHR-P assessment. The alternative approach of using CHR-P instruments to screen all individuals accessing secondary mental health care is logistically untenable (5). These limitations of knowledge can be tackled through a refined approach for the detection of at-risk individuals and the prediction of psychosis. Recent studies have developed and externally validated individualized risk prediction tools that depend on few established risk factors for psychosis (34–36), with the ultimate goal of improving the detection of at-risk cases. This line of research can be further expanded through the integration of recent epidemiological research on genetic risk factors, demographic and environmental risk factors for psychosis.

### Implications for Neuroscience and Behavioral Research

The above limitations have a profound impact on neurobiological research conducted in CHR-P samples. Idiosyncratic recruitment strategies lead to uncontrolled accumulation of risk and protective factors and increase the clinical heterogeneity of CHR-P samples (33). In turn, the high clinical heterogeneity has hampered the discovery of reliable and replicable biomarkers of psychosis risk (21). As summarized in Figure 3, CHR-P samples that had been largely recruited through the community (37) showed a dilution in pre-test risk (14) with a resulting lack of gray matter abnormalities, when compared to controls (28). Because of these issues, no reliable neuroimaging, electrophysiological or neurocognitive biomarker of psychosis risk has been validated for clinical use in CHR-P samples yet. Furthermore, the limited detection power of the current recruitment strategies adds concerns, undermining the assumption that the neurobiological alterations reported in CHR-P individuals would represent prototypical features preceding the onset of psychosis (3).

## The Example of Polygenic Risk Score

High heritability of psychotic disorders, such as schizophrenia, indicates a substantial impact of inherited genetic variants on risk. Although genetic variants can be common or extremely rare, nearly one-third of the genetic risk of schizophrenia is indexed by common alleles genotyped through arrays in genome-wide association studies (GWAS) (38). As each marker individually explains only a small proportion of the genetic variation, recent research has developed polygenic risk scores in order to examine disorder prediction by genetic variants “en masse,” summarizing risk variants across many associated loci into quantitative scores (39). Such an approach requires robust a priori knowledge on the association between specific loci and psychosis as a first step (38). The polygenic risk score was therefore grounded on the GWAS meta-analysis conducted by the Schizophrenia Working Group of the Psychiatric Genomics Consortium (38). This meta-analysis identified that despite the small effect sizes of single loci, the cumulative effect of thousands of schizophrenia-associated loci expressed a polygenic risk score explained up to 18% of variance between cases of schizophrenia and controls in GWAS studies and 7% of the variance on the underlying liability scale to schizophrenia in the general population (38). Polygenic risk scores have been used to predict case-control status at the time of a first episode psychosis, explaining nearly 9% of variance (39). However, as heritability of schizophrenia is 64% (95%CI: 62–68%) (40), a large proportion of the variance remains unaccounted. As the variance explained is too small for individual risk prediction, the use of polygenic risk scores in clinical routine is currently insufficient on its own (38, 41).

## Toward a Polyrisk Score Encompassing Non-genetic Risk/Protective Factors

Given the small proportion of variance explained, risk prediction needs to be boosted by supplementing the polygenic risk scores with additional information. The model that has received some empirical support indicates that the etiology of psychotic disorders like schizophrenia involves direct genetic and environmental effects, along with their interaction (42, 43). In reality, some of the most predictive factors, including family history of mental illness and socioeconomic status, include both a genetic and environmental component and hence a distinction between genetic and environmental factors may be spurious. We will, therefore, adopt a pragmatic approach and use the term non-genetic to define sociodemographic, social, parental, perinatal, later risk or protective factors, or antecedents -see below-. The use of a priori clinical knowledge is a robust method for developing a clinical prediction model [for a review on this see (44)].

### Definition of Risk and Protective Factors for Psychosis

**Figure 4:**
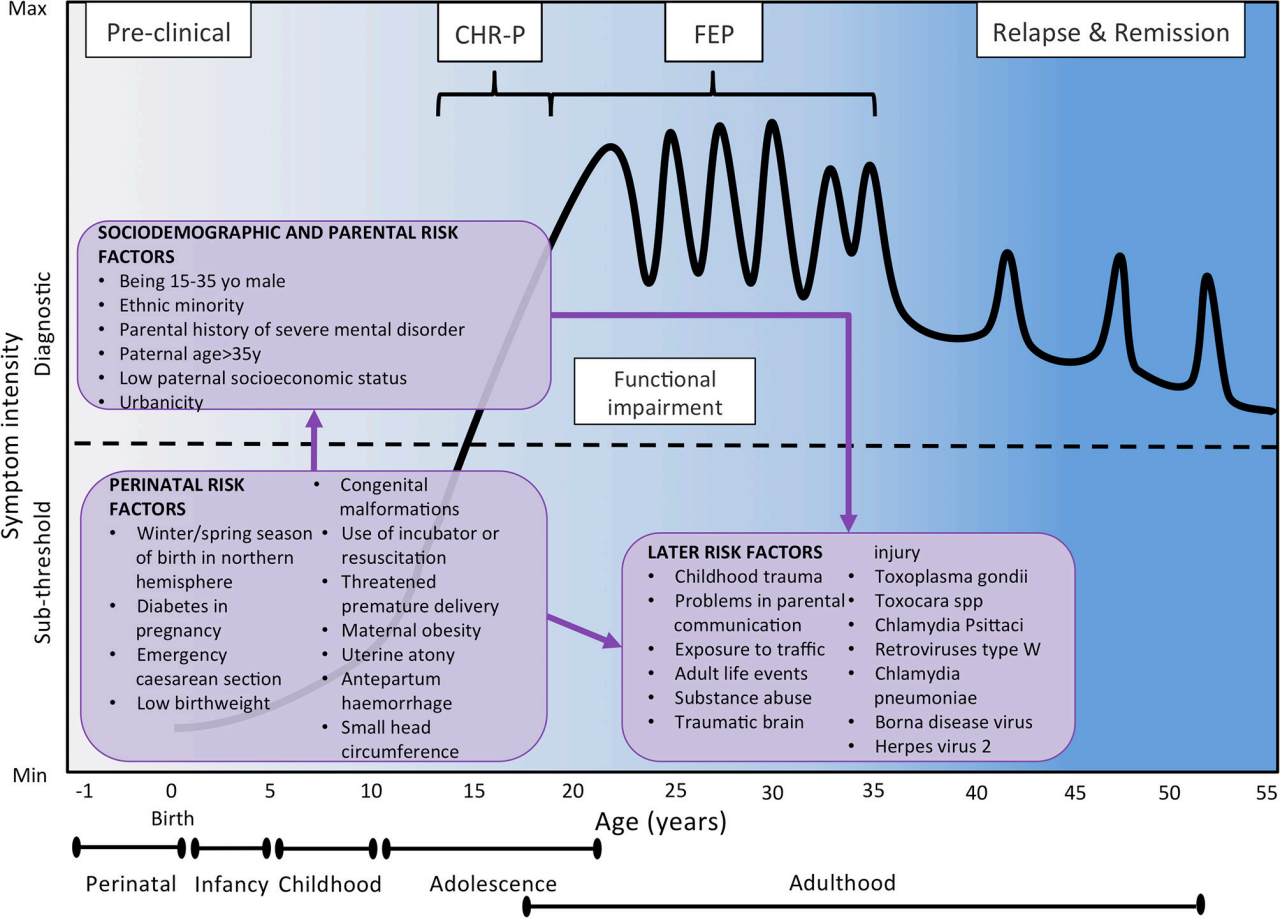
Putative model of the onset and progression of psychosis in relation to non-purely genetic risk factors and developmental processes affected by the disorder. Sociodemographic and parental risk factors and perinatal risk factors have been implicated during the preclinical phase, usually observed from the birth to infancy, childhood and early adolescence. Additional later factors occurring during later adolescence and early adulthood can trigger the onset of attenuated psychotic symptoms, functional impairment and help-seeking behavior, which constitute the CHR-P stage. The diagnosis of psychosis, which operationally corresponds to the first episode of psychosis, is usually made during the adolescence or early adulthood, with a peak from 15 to 35 years (48). Once diagnosed, psychosis usually follows a fluctuating course punctuated by acute exacerbation of psychotic crises superimposed upon a background of poorly controlled negative, neurocognitive, and social cognitive symptoms. The pink boxes represent the risk factors for psychosis as identified by the umbrella review (48). There is no assumption that these risk factors are of causal nature or that they are independent of each other. Furthermore, certain risk factors may actually represent outcomes of earlier risk factors. Figure based on the data reported in Fusar-Poli et al. (16). FEP, First Episode Psychosis; CHR-P, Clinical High Risk for Psychosis.

For descriptive purposes, in the current manuscript risk/protective factors for psychotic disorders are grouped across domains previously defined: sociodemographic and parental factors, perinatal factors, later factors, and antecedents (45–47). Demographic, parental, social, and perinatal risk factors are generally believed to exert their role during the early developmental phases that precede the onset of psychosis (see also Figure 4). On the contrary, later risk factors and antecedents are believed to modulate psychosis risk in the post perinatal period, from late childhood up to the phases that shortly precede the onset of a psychotic disorder. While later risk factors would indicate a passive exposure to socio-environmental factors, antecedents would index premorbid deviations in functioning and developmental milestones and active risk-modifying processes involved in psychosis onset (45–47). However, the boundaries of these categories may in fact overlap.

### Evidence and Classification of Risk and Protective Factors for Psychosis

The inclusion of non-genetic factors in the development of polyrisk scores is not a conceptually novel approach, but it has been limited to date by the lack of established and robust a priori knowledge on the association of non-genetic factors and psychotic disorders. Such a limitation has been recently overcome by an umbrella review, which is a meta-analysis of meta-analyses or reviews, investigating several non-genetic risk/protective factors of psychosis that operate at an individual level. The umbrella review further classified these factors into convincing (class I), highly suggestive (class II), suggestive (class III), weak (class IV), and non-significant (ns) evidence, according to a standardized classification already widely adopted in other branches of clinical medicine (48) to control for potential biases.

**Figure 5:**
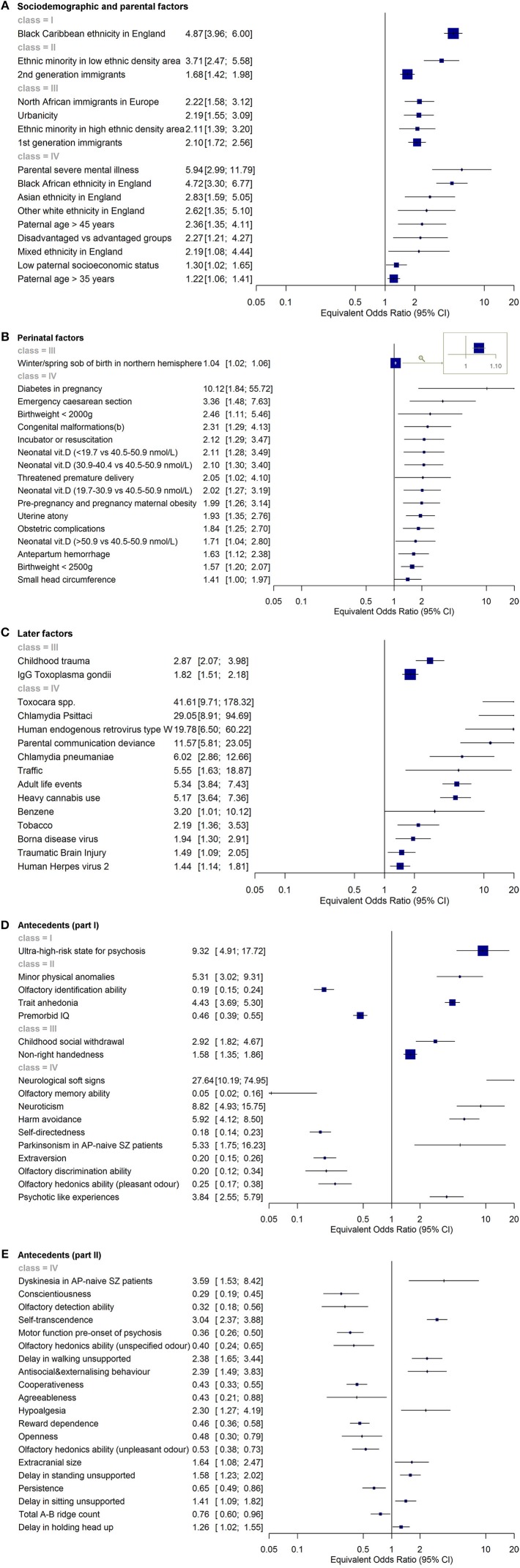
**(A–D)** Umbrella review (meta-analysis of published meta-analyses or systematic reviews published up to January 31, 2017) investigating the level of evidence for an association of sociodemographic and parental **(A)**, perinatal **(B)**, later **(C)** risk/protective factors and antecedents **(D,E)** and psychotic disorders. Each of these factors operate at the individual level. Incidence rate ratio (IRR), odds ratio (OR), risk ratio (RR), greater than one or standardized mean difference (Hedges' g for continuous measures) greater than zero indicated that the factor was associated with an increased likelihood of psychotic disorders. IRR, OR, and RR lower than 1 or Hedges' g lower than zero indicated that the factor was associated with a reduced likelihood of psychotic disorders, i.e., it was protective. The level of evidence is further stratified according to established criteria in different classes: convincing (class I), highly suggestive (class II), suggestive (class III), weak (class IV), and non-significant (ns) evidence. The figures are based on the data from Radua et al. (48).

For instance, sensitivity analyses restricted to prospective studies assessed whether there was evidence for risk factor pre-existing before disorder onset, therefore controlling for reverse causation (48). By providing the required gold-standard *a priori* knowledge (44), the core results of this meta-analysis (Figures 4, 5A–E) place the groundwork for the development of a comprehensive polyrisk score for psychosis prediction.

#### The Substantial Role of Sociodemographic Risk/Protective Factors

Most aetiopathogenic models for psychotic disorders have focused on genetic and environmental risk factors, while demographic factors have been investigated to a lesser degree, presumably in the light of the fact that these factors are not strictly modifiable. Nevertheless, the recent umbrella review found a main effect for male gender, a main effect for 15–35 years of age (48) and an association between psychotic disorders and being a male aged 15–40 year-old (48). Age older than 35 was found to be a protective factor (48). The additional risk factor that was consistently associated with psychosis was ethnicity, variously defined as being an ethnic minority or as having an immigrant status or through specific categories of ethnicity. For instance, being of a black Caribbean (OR 4.87, class I), black African (OR 4.72), Asian (OR 2.83) or mixed (OR 2.19) ethnicity in England or North African in Europe (OR 2.22) was associated with an increased liability to psychosis (48). These findings are of significant value for the development of polyrisk scores as they suggest that these factors should always be assessed and considered for the prediction of psychosis onset. In other branches of medicine, age and gender are consistently used in individualized risk scores for predicting cardiovascular diseases (QRISK) (49), diabetes (AUSDRISK) (50) or stroke (CHA2DS2-VASc score) (51). Recent confirmation of the clinical utility of demographic variables for predicting psychosis onset was shown by a recent study that included age, gender, age by gender, and ethnicity in an individualized risk estimation tool for predicting psychosis in secondary mental health care (34).

#### Parental and Perinatal Risk/Protective Factors

Psychotic syndromes are disorders of adapting to the environment (52), which include parental, perinatal, later risk factors, along with antecedents. The umbrella review identified that parental factors such as paternal age (>35 OR 1.22, >45 OR 2.36), low paternal socioeconomic status (OR 1.30) and parental history of severe mental disorder (OR 5.94) were all associated with psychosis (48). Polygenic studies controlling for the effect of parental risk factors found that parental socioeconomic status accounted for 45.8% (95%CI, 36.1–55.5) of cases with schizophrenia (53). Assuming social causation, this indicates that the impact of the environment is actually higher than the genetic factors. Similarly, a recent study indicated that polygenic risk scores can improve their predictive value, explaining 17.4% variance if used in cases with a family history of schizophrenia/psychoses (i.e., prediction by PRS including more genetic variants) (53). These findings concur with the need for integrating genetic and parental risk factors for psychosis in a polyrisk score. Some studies have already supplemented the polygenic score profile with information on family history for psychotic disorders (54). Other risk factors could be considered for the development of a polyrisk assessment including urbanicity (OR 2.19) (48). As this factor was robust and survived sensitivity analyses (class I), it should always be measured and considered in polyrisk assessment approaches (48). Finally, a series of perinatal risk factors were shown to be useful for the polyrisk score. The most robust of them was winter/spring season of birth in northern hemisphere (OR 1.04, class III) (48), followed by diabetes in pregnancy (OR 10.12), emergency cesarean section (OR 3.36), low birth weight (<2000 OR 2.46, <2500 OR 1.57), congenital malformations (OR 2.31), use of incubator or resuscitation (OR 2.12), threatened premature delivery (OR 2.05), maternal obesity (OR 1.99), uterine atony (OR 1.93), antepartum hemorrhage (0.163), and small head circumference (OR 1.41) (48). To the best of our knowledge, no studies have attempted to combine polygenic risk assessment with these risk factors, and this may prove to be a promising avenue of research.

#### Later Risk/Protective Factors

Later risk factors that have been associated with psychosis include a variety of environmental risk factors such childhood trauma (OR 2.87), problems in parental communication (OR 11.57), exposure to traffic (OR 5.55), adult life events (OR 5.34), substance abuse such as heavy cannabis (OR 5.17), benzene (OR 3.20) or tobacco (OR 2.19), and traumatic brain injury (OR 1.49) (48). Later risk factors also include a series of infective agents such as IgG Toxoplasma gondii (OR 1.82), Toxocara (OR 41.61), Chlamydia Psittaci (OR 29.05), retroviruses type W (OR 19.78), Chlamydia pneumoniae (OR 6.02), Borna disease virus (OR 1.94), and herpes virus 2 (OR 1.44) (48). Exposures to childhood trauma and Toxoplasma gondii were most robustly associated with increased risk of psychosis (class III), while the other later factors showed weak association (48).

#### Antecedents

There are numerous antecedent factors associated with psychosis. The risk factor with the most robust evidence was CHR-P status (OR 9.32, class I), followed by minor physical anomalies (OR 5.30), trait anhedonia (OR 4.41), olfactory identification ability (OR 0.19) and premorbid IQ (0.47) (all class II) (48). Childhood social withdrawal (OR 2.91) and non-right handedness (OR 1.58) were also associated with increased risk of psychosis with other antecedent factors showing weak association (48).

## Methodological Considerations for the Development of a Psychosis Polyrisk Score (PPS)

### Specificity, Universality and Durability of Non-genetic Risk Factors

A crucial step toward the development of a PPS is to deconstruct and standardize the specificity of non-genetic risk factors. While polygenic risk scores build on variation in specific single nucleotides in exact positions in the genome, and thus are unambiguously defined at all ages for all individuals and thus across all studies, specificity of most non-genetic risk factors is not completely determined. For example, some of them may be ascertained through a multitude of instruments of questionable comparability. Others may require contextual specifiers (e.g., Black Caribbean Ethnicity in England), since their predictive validity may depend on their universality in different cultural scenarios. More on this point, other factors may be influenced by changes in the contextual environment (e.g., socioeconomic status) and therefore their durability over time periods may be questionable. An additional problem is that many factors are affected by both genetic and non-genetic influences; therefore the specific components of these risk factors should also be better elucidated. For instance, the effect of parental history of schizophrenia/psychoses is only partly mediated through the individual's genetic liability (54). The impact of shared environmental influences in the context of the parental history of severe mental illness on liability to schizophrenia amounts to nearly 11% (55). The umbrella review has adopted a pragmatic approach to partially mitigate the above concerns. First, it included several meta-analyses that were conducted worldwide and that were representative of different contextual environments (universality). These studies were also published over two decades, minimizing the confounding role of time (durability). Finally, the umbrella review indicated that despite heterogeneous measurements (specificity) and spurious risk factors (encompassing genetic and non-genetic components), the factors analyzed were robustly associated with psychosis onset.

### Assessment of Factors

The concurrent assessment of several demographic and environmental risk factors for psychosis listed in Figures 5A–E may appear logistically unviable in clinical practice. However, it would be facilitated by a sequential testing procedure (56). For instance, all demographic and parental risk/protective factors, as well as some environmental (urbanicity, winter/spring season of birth) and later risk factors (adult life events, tobacco use, cannabis use, childhood trauma, traffic) can be self-administered or automatically extracted from electronic medical records or from geolocating apps that capitalize on recent e-Health advancements. For the individuals whose predicted polyrisk of psychosis is over a certain threshold, a clinical comprehensive polyrisk assessment can be then performed in a sequential fashion (56). Such an assessment may involve more accurate testing to collect the remaining risk factors—blood sampling for assessing the exposure to infective agents as well as to estimate the polygenic risk, consultation of obstetric records or by interviewing the patients' relatives and clinical interviews.

### Developmental Challenges of the PPS

The PPS can be subsequently developed for reproducing the methodology employed to get the polygenic risk score, based on an additive model for quantifying an individual's genetic loading for a disorder, as conferred by multiple risk alleles (57). From a statistical perspective, polygenic scores are weighted sums of the genotypes of a set of variants. To develop a PPS, the presence or absence of each of the above risk factor should be determined for each individual. The log of the odds ratio for each risk factor listed in Figures 4, 5A–E can subsequently be multiplied by either 1 (risk factor deemed present in the individual) or 0 (risk factor deemed absent). These products can successively be added together and the sum divided by the total number of risk factors assessed (54). Validation of this approach through a prospective longitudinal study would be a key stage of the development of such a tool. Furthermore, since some of the factors are mutually exclusive or may be correlated some pruning may be required to reduce redundancy. An additional problem may be that missing values such as not knowing family history in adopted individuals should be considered and potentially imputed with statistical methods.

## Psychosis Polyrisk Score (PPS) Prototype

In the second part of this review we will apply the concepts developed above to operationalize a PPS prototype.

### Development and Operationalization of the PPS

To attain the most robust prognostic tool, the umbrella review factors were used. Factors with the greatest strength of evidence (class I–III) were initially considered for the PPS. Since our aim was to improve the detection of individuals at-risk for psychosis at scale, logistical considerations were of paramount importance. We thus applied a pragmatic filter to exclude factors that could not easily be measured at scale (such as Toxoplasma Gondii IgG). A total number of 13 class I–III factors that can be pragmatically measured were included in the prototype PPS assessment. To ensure accurate scoring, appropriate measurement and cut-offs for each factor is of great importance. Where possible, the same tools were selected to assess the presence of factors as used in their respective meta-analyses in the umbrella review (48). This was similarly true for cut-offs to preserve the validity of the Risk Ratios. The list of included factors, along with their definitions and the tentative cut-offs for defining each respective Risk Ratio can be seen in Table 1. While this may not be the most predictive set of factors in existence, one of the major characteristics of the PPS is that it is optimizable i.e., it can be refined by the inclusion of other predictors or by the fine tuning of the cut-offs to be used.

**Table 1:** Operationalization of factors in the Psychosis Polyrisk Score (PPS).

**Factor**	**Operationalization**	**Pilot cut-offs**
Childhood trauma	Childhood trauma questionnaire	Moderate to severe
Ethnicity	Self-defined	Non-white ethnicity
Immigration	Self-defined	First- or second-generation
Premorbid IQ	National adult reading test	<93.6
Non-right handedness	Self-defined	Non-right handedness
Olfactory identification ability	University of Pennsylvania smell identification test	Mild microsmia
Clinical High Risk state for Psychosis	Prodromal questionnaire (16-item version)	>9
Urbanicity	Population density of local authority	Living in local administrative unit (LAU) where the majority of the population lives in an urban center of at least 50,000 inhabitants

The PPS, similar to PRS, involves a weighted sum of exposure to risk and protective factors, using the relative risks associated with each factor [seen in (48)]. To construct the PPS we first estimated a raw score for each factor as the 10-base logarithm of its relative risk. For example, the estimated relative risk of psychosis in individuals living in urban settings is 2.2, and thus the raw score of the urbanity factor was log_10_(2.2) = 0.34. We then subtracted the population average of this raw score, so that individuals at-risk would have positive scores and the remaining individuals would have negative scores, with an average of zero. For example, given that ~73.6% individuals live in urban settings (and thus 26.4% in rural settings with a raw score of 0), the population average of the urbanicity factor should be (73.6% × 0.34) + (26.4% × 0) = 0.25. We subtracted this average from the raw scores, i.e., the subtracted score was 0.34–0.25 = 0.09 for individuals in urban settings and 0–0.25 = −0.25 for individuals in rural settings. Further information about prevalence data used can be seen in Table S1. Finally, for the ease of use we multiplied the subtracted scores by 10 and rounded them to the nearest half integer. In the example, the final scores were 0.09 × 10 ≈ 1 for individuals in urban settings and −0.25 × 10 = −2.5 for individuals in rural settings. The final scoring of the PPS is reported in Table 2.

**Table 2:** Scoring system for the Psychosis Polyrisk Score (PPS).

**Factor**			**PPS**
Childhood trauma	Yes		4
	No		−0.5
Ethnicity	White		−2
	Black Caribbean	In low ethnic density area	6
		In medium ethnic density area	5.5
		In high ethnic density area	3.5
	Other	In low ethnic density area	3.5
		In medium ethnic density area	3
		In high ethnic density area	1
Immigration	Not immigrant		−0.5
	1st gen immigrant	From North Africa	3
		From other regions	2
	2nd gen immigrant	From North Africa	2.5
		From other regions	1.5
Premorbid IQ	<93.6		2
	>93.6		−1
Non-right handedness	Yes		2
	No		0
Olfactory identification ability	Yes		5.5
	No		−1.5
Clinical high risk state for psychosis	>9		8.5
	<9		−1.5
Urbanicity	Yes		1
	No		−2.5

*Please see Table 1 for the operationalisation of these predictors*.

Furthermore, some adaptations were introduced to mitigate for conceptual dependency across some factors. Factors related to immigration had logical dependencies between them, i.e., immigrants cannot be both first-generation and second-generation, and North African immigrants are first- or second-generation immigrant. We combined these factors following this logic and assuming that the proportion and extra risk of North African immigrants is similar in first- and second-generation immigrants (58). Factors related to ethnicity had similar logical dependencies between them, i.e., black Caribbean is a non-white ethnicity, and individuals cannot be from a low ethnic density area, from a medium density area and from a high ethnic density area at the same time. We combined these factors again following this logic and assumed that the proportion and extra risk of black Caribbean individuals between non-white ethnicity individuals is similar in low, medium and high ethnic density areas.

### Simulating the PPS Scores in the Hypothetical General Population

**Figure 6:**
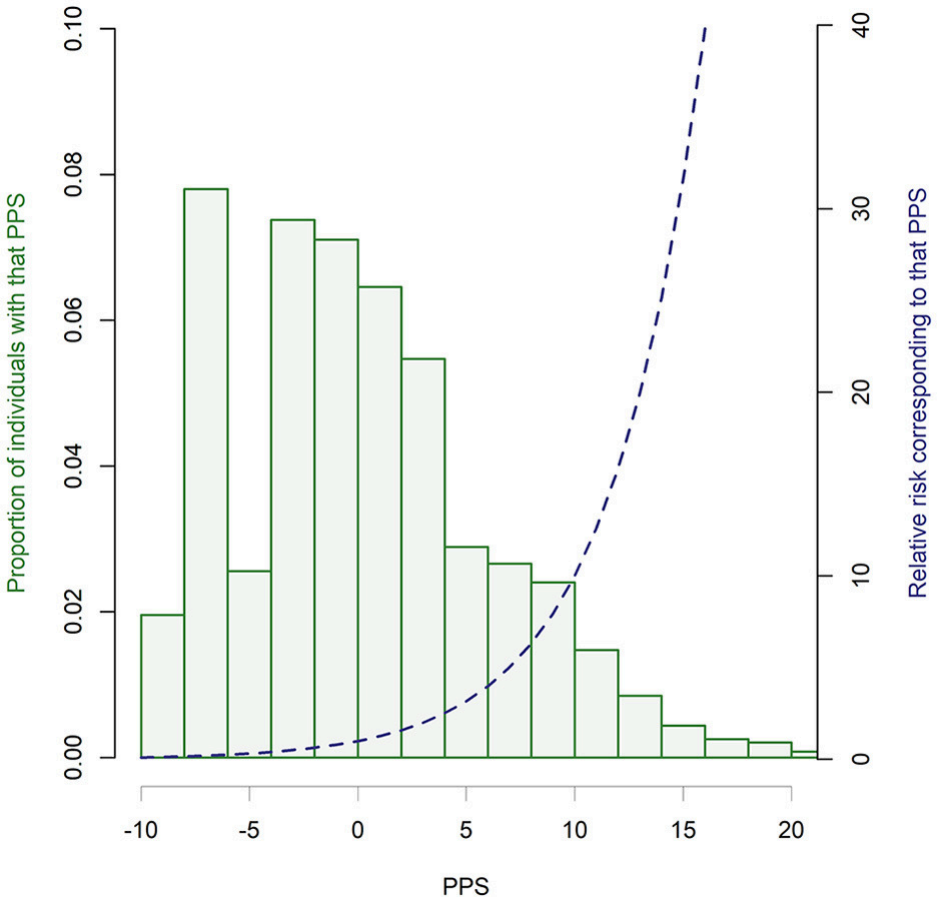
Distribution of PPS scores in a hypothetical general population. Histogram bars indicate the proportion of individuals receiving each PPS score (in 0.5 increments) based on the prevalence of risk factors and 10,000,000 permutations. Blue dotted line illustrates the equivalent relative risk for PPS scores.

As indicated in Table 2, an individual's potential PPS score ranges between−7.5 (least psychosis risk) and 32 (greatest psychosis risk). Utilizing prevalence data for each risk factor (Table S1), we ran 10,000,000 permutations to investigate the range and distribution of PPS scores in the general population. While this does require external longitudinal validation, this is the first attempt to do this in the field. As illustrated in Figure 6, the distribution is skewed to the left with 53.6% of individuals having a negative PPS score (RR < 1), and a further 25.7% with PPS scores between 0 (RR = 1) and 5 (RR = 3). This leaves only 21.6% with RR >3, with only 1.8% having an RR >30.

## Combination of the PPS With Polygenic Risk Scores: Potentials and Challenges

### Integrating the Genetic and Non-genetic Components

Consequently, the above-described PPS mostly includes non-genetic risk factors. Therefore, it can be integrated with the genetic risk score acquired in the same individuals. Integration of genetic and non-genetic information may benefit from considering gene by environment interactions. There is no consensus on the most effective model. The original GWAS meta-analysis found no epistatic or non-additive effects between the candidate loci (38) and other studies did not find interactions between polygenic risk score and environmental risk factors (53, 59). On the other hand, an interaction between polygenic risk score and demographic factors is demonstrated in individuals of African ancestry (poor prognostic accuracy) (39) or with a family history of psychosis (high prognostic accuracy) (60). Since the vast majority of potential interactions across genetic and non-genetic risk have not been tested yet (38), at present, an additive model that sums all known genetic and non-genetic risks is a pragmatic approximation. An additive approach combined with weighted summation to account for interactions has recently shown promise (61). A recent review of gene by environment interactions confirmed that polymorphisms of catechol-O-methyltransferase (COMT), brain-derived neurotrophic factor (BDNF), and FK506-binding protein 5 (FKBP5) genes might interact with early life stress and cannabis abuse or dependence, influencing various outcomes of schizophrenia spectrum disorders (62). In the future, robust gene by environment interactions can be incorporated in the same way as other combinations of risk factors were already incorporated in the umbrella review. This would be facilitated by the proposed comprehensive approach that assesses several candidate risk factors and analyses them in a multivariate fashion. While this would be the ideal target for advancing the development of these integrated scores, with the evidence currently available to us, the most pragmatic approach would be an additive model.

### Prognostic Modeling Challenges

The development and validation of a comprehensive genetic and non-genetic polyrisk score is faced by some prognostic modeling challenges. It is important to highlight that the association measures reported by the umbrella review were based on a univariate meta-analysis. Therefore, there is no assumption that the reported risk or protective factors are independent, and they could be mutually confounded. For instance, in the case of a parental history of severe mental disorder and paternal socioeconomic status the former could confound the impact of the latter, or conversely, low socioeconomic status may lead to certain mental disorders. In contrast, the polygenic risk score is based on genetic variations that are far apart in order to avoid linkage disequilibrium. Future studies are therefore requested to measure multiple exposures in the same individuals, to clarify the independence of each exposure. This should also be facilitated by data sharing policies across ongoing studies that would allow performing patient-data meta-analyses or umbrella reviews. Availability of advanced statistical learning methods (e.g., random forests, vector support machines, penalized linear regression methods) could also help to create risk prediction algorithms for complex multivariate situations in which multiple collinear risk factors are involved (63). A related problem is that the reported associative measures were all estimated in the same pool of meta-analyses. Although the sample size was the largest to date, and the evidence was subjected to established classification criteria, no strict external validation in an independent dataset was performed. As a result, PPS created on the basis of the measures reported in the umbrella review should be validated in independent datasets to test their actual prognostic performance (44).

## Clinical Potential and Future Research

While the next decade of research will be requested to address the above challenges, the PPS approach holds promise for resolving the weaknesses of the CHR-P paradigm as well as to overcome knowledge in the etiology of psychotic disorders.

### Clinical Staging and Dynamic Mapping of Developmental Risk Trajectories

**Figure 7:**
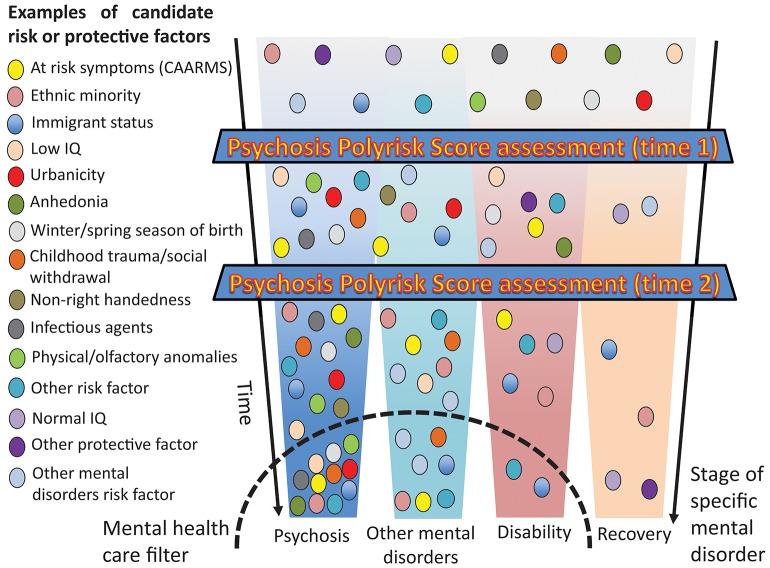
Putative PPS assessment for the detection of at-risk individuals and the prediction of psychosis. Risk or protective factors that are diluted during the pre-clinical stages may accumulate as the individual progresses across different stages until they trigger signs or symptoms and functional impairment that are associated with help-seeking behavior and access to mental health care. In the later stages, specific aggregations of risk and protective factors may be associated with specific clinical outcomes.

The PPS approach combined with a polygenic risk score would allow researchers to control and replicate CHR-P risk enrichment in a controlled manner, while at the same time facilitating identification of at-risk cases on the basis of a determinate accumulation of risk factors. This would improve the detection of at-risk case and refine the prediction of psychosis. Furthermore, as illustrated in Figure 7, the PPS assessment accommodates a clinical staging framework for the development of psychosis, which has recently been reviewed elsewhere (3). For this aim, it will be important to draw a distinction between individually stable factors (genes, prenatal, and early childhood) that can be carried forward and developmental/state factors that will require reassessments over the life course. For instance, the PPS assessment can potentially be administered during the preclinical phase in non-clinical samples, such as screening programmes for schools or non-help-seeking youths in the community (time 1) for identifying at-risk groups and facilitate selective preventative focused interventions (3). Such an assessment can be followed by testing (56) in individuals who present with subtle symptoms of psychosis-like CHR-P features in the ones accessing secondary mental health services. Child and adolescent mental health services and early intervention services may be particularly suited for such an assessment (60) (Figure 7). The systematic incorporation of a temporal dimension (64) in the polyrisk assessment is consistent with a developmental framework for mental disorders that has recently been recommended for advancing etiological knowledge (65). Our group is currently piloting a beta version of the PPS after individuals are identified to be at-risk for psychosis by a validated transdiagnostic risk calculator (34–36).

### Transdiagnostic Potential for the Prediction of Non-psychotic Mental Disorders

There is emerging evidence that the same risk factors may be associated with multiple types of disorders, beyond psychosis (pleiotropy). For instance, another recent umbrella review has indicated that childhood adversity, exposure to *Toxoplasma gondii* and a history of head injury are also linked to bipolar disorders (66). These findings do not eliminate the possibility that even if these risk factors are shared between bipolar disorder and psychosis, the loading and combination of factors that results in either of the two disorders may still be constituted of unique dimensions (65). While the risk factors themselves may be shared with other psychiatric disorders, the weighting of these factors will be different i.e., the same factor could have a differential impact on risk for different disorders. What is evident is that there is great potential for transdiagnostic research that focuses on broad and heterogeneous samples of mental disorders. Unfortunately, to date, transdiagnostic research has been poorly operationalized and has not provided robust evidence to improve the current classification system (67).

### The Role of Biomarkers

In the current perspective, we selectively focused on genetic and non-genetic factors, while biomarkers were not primarily discussed. One of our aims was to improve the modest detection power of the CHR-P paradigm and the use of biomarkers would present specific challenges that would require a separate manuscript. For example, risk stratification models that include neuroimaging, electrophysiological, or peripheral biomarkers (68–70) have been mostly developed and validated within CHR-P samples (56). Therefore, these models could not be used to improve the detection of at-risk individuals. Furthermore, their broader use in the community or National Health Service scenarios is hampered by feasibility and economic caveats, because these models are logistically complex. Our group has recently demonstrated that risk stratification models encompassing neuroimaging, electrophysiological and peripheral biomarkers could rather be used in subsequent testing, in line with similar stepped risk enrichment assessments that are used in clinical medicine (56).

## Conclusions

The combination of risk/protective factors encompassing genetic (PRS) and non-genetic information (PPS) holds promise for overcoming the epidemiological weakness of the CHR-P paradigm. The PPS conceptually and empirically developed here will facilitate future research in this field and hopefully advance our ability to detect individuals at-risk for psychosis and forecast their clinical outcomes.

## Ethics Statement

This study was supported by the King's College London Confidence in Concept award from the Medical Research Council (MRC) (MC_PC_16048) to PF-P. This study also represents independent research part funded by the National Institute for Health Research (NIHR) Biomedical Research Centre at South London and Maudsley NHS Foundation Trust and King's College London. The views expressed are those of the author(s) and not necessarily those of the NHS, the NIHR or the Department of Health and Social Care. The funders had no influence on the design, collection, analysis and interpretation of the data, writing of the report and decision to submit this article for publication.

## Author Contributions

PF-P conceived the study under the supervision of RU. DO acquired the data. AR and JR coordinated the statistical analysis. PF-P and DO drafted the manuscript, and AR, JR, and RU made substantial contribution to the revision of the initial manuscript and interpretation of data.

### Conflict of Interest Statement

The authors declare that the research was conducted in the absence of any commercial or financial relationships that could be construed as a potential conflict of interest.
