# Supplementary material for: Psychosis Polyrisk Score (PPS) for the Detection of Individuals At-Risk and the Prediction of Their Outcomes
Source: Front Psychiatry. 2019 Apr 17;10:174. doi: 10.3389/fpsyt.2019.00174 (PMC6478670; doi:10.3389/fpsyt.2019.00174)
Supplement: Supplementary file 1 [file Table_1.docx]

**Supplementary Online Content**

Oliver D, Radua J, Reichenberg A et al. Psychosis polyrisk score (PPS) for the detection of individuals at-risk and the prediction of their outcomes

**Table S1** - Prevalence of factors used in PPS in the general population

**References**

| **Table S1** Prevalence of factors used in PPS in the general population | | |
| --- | --- | --- |
| **Factor** | **Prevalence** | **Prevalence source** |
| Clinical high risk state for psychosis | 0.13936 | Sensitivity/specificity of Prodromal Questionnaire (PQ) (1)  Prevalence of UHR (2) |
| Black Caribbean ethnicity in England | 0.03462 | 2011 UK Census (3) |
| Olfactory identification ability | 0.24 | University of Pennsylvania Smell Identification Test (4) |
| Ethnic minority in low ethnic density area | 0.2085 | Mean % non-white ethnicity in lowest 25% ethnic density areas (3) |
| Premorbid IQ | 0.3348111 | Corresponds to <= 93.6 if mean=100 and sd=15 (5) |
| 2nd generation immigrant | 0.092 | Eurostat LFS 2014 (6) |
| Childhood trauma | 0.117560497 | Mean of sexual, physical, emotional and psychological abuse, pooled from controls, false positives (FP) and true positives (TP) (7) |
| North African immigrants in Europe | 0.000023 | Annual Population Survey 2018 (8) |
| Urbanicity | 0.736 | United Nations, Department of Economic and Social Affairs (9) |
| Ethnic minority in high ethnic density area | 0.6799 | Mean % non-white ethnicity in top 25% ethnic density areas (3) |
| 1st generation immigrant | 0.168 | Eurostat LFS 2014 (6) |
| Non-right handedness | 0.059 | Finnish twin cohort study (n=30,161) (10) |
| Seasonality of birth in Northern hemisphere | 0.5 | N/A |

**References**

1. H. K. Ising, W. Veling, R. L. Loewy, M. W. Rietveld, J. Rietdijk, S. Dragt, R. M. Klaassen, D. H. Nieman, L. Wunderink, D. H. Linszen and M. van der Gaag: The validity of the 16-item version of the Prodromal Questionnaire (PQ-16) to screen for ultra high risk of developing psychosis in the general help-seeking population  *Schizophrenia Bulletin*, 38(6), 1288-96 (2012) doi:10.1093/schbul/sbs068

2. B. G. Schimmelmann, C. Michel, A. Martz-Irngartinger, C. Linder and F. Schultze-Lutter: Age matters in the prevalence and clinical significance of ultra-high-risk for psychosis symptoms and criteria in the general population: Findings from the BEAR and BEARS-kid studies. *World Psychiatry*, 14(2), 189-197 (2015)

3. O. f. N. Statistics, N. R. o. Scotland and N. I. S. a. R. Agency: 2011 Census aggregate data. In: UK Data Service, (2016) doi:10.5257/census/aggregate-2011-1

4. R. L. Doty: University of Pennsylvania Smell Identification Test Administration Manual. Sensonics International, New Jersey, USA (2013)

5. G. M. Khandaker, J. H. Barnett, I. R. White and P. B. Jones: A quantitative meta-analysis of population-based studies of premorbid intelligence and schizophrenia  *Schizophrenia Research*, 132(2-3), 220-227 (2011) doi:10.1016/j.schres.2011.06.017

6. Eurostat: European Union Labour Force Survey. In, (2014)

7. M. van Nierop, J. van Os, N. Gunther, I. Myin-Germeys, R. de Graaf, M. ten Have, S. van Dorsselaer, M. Bak and R. van Winkel: Phenotypically Continuous With Clinical Psychosis, Discontinuous in Need for Care: Evidence for an Extended Psychosis Phenotype. *Schizophrenia Bulletin*, 38(2), 231-238 (2012) doi:10.1093/schbul/sbr129

8. O. o. N. Statistics: Annual Population Survey. In, (2018)

9. U. N. D. o. E. a. S. A. P. Division: World urbanisation prospects. In, (2014)

10. E. Vuoksimaa, M. Koskenvuo, R. J. Rose and J. Kaprio: Origins of handedness: a nationwide study of 30,161 adults  *Neuropsychologia*, 47(5), 1294-301 (2009) doi:10.1016/j.neuropsychologia.2009.01.007
